# PEGylation in Pharmaceutical Development: Current Status and Emerging Trends in Macromolecular and Immunotherapeutic Drugs

**DOI:** 10.7759/cureus.66669

**Published:** 2024-08-12

**Authors:** Karthick R Santhanakrishnan, Jebastin Koilpillai, Damodharan Narayanasamy

**Affiliations:** 1 Pharmacy, Sri Ramaswamy Memorial (SRM) Institute of Science and Technology, Chennai, IND; 2 Pharmaceutics, Sri Ramaswamy Memorial (SRM) Institute of Science and Technology, Chennai, IND

**Keywords:** recent approved drugs, novel treatments, reticuloendothelial system clearance, macromolecular drugs, pegylation

## Abstract

The purpose of the research is to examine the advantages and difficulties of target-site drug delivery methods, with an emphasis on the application of polyethylene glycol (PEG) to enhance drug solubility, bioavailability, and immune response characteristics. It has been demonstrated that this method lowers immunogenicity, enhances pharmacokinetics, and helps drugs pass the blood-brain barrier while reducing reticuloendothelial system clearance. PEG and its derivatives are being used more and more to alter therapeutic substances, offering an escape from some of the drawbacks of conventional medication formulations. In the review, different PEGylation tactics are examined, including cutting-edge methods for reversing multi-drug resistance in nanocarriers. PEGylation has a number of benefits, but there are still drawbacks, including the immunogenic reaction to PEG, which is sometimes referred to as "anti-PEG antibodies," and stability problems that call for the creation of countermeasures. The study devotes a large amount of its space to listing FDA-approved PEGylated medications, emphasizing their therapeutic advantages and clinical uses in a range of medical specialties. The research also explores the regulatory environment that surrounds PEG, closely examining its effectiveness and safety in medication compositions. The review goes beyond PEGylation and includes lipid-based nanocarriers, including liposomes, nanostructured lipid carriers (NLCs), and solid lipid nanoparticles (SLNs). Because these nanocarriers can target specific tissues or cells, improve bioavailability, and encapsulate pharmaceuticals, they are becoming more and more significant in drug delivery systems. The Target Product Profile (TPP) and Quality by Design (QbD) principles serve as the foundation for the creation and characterization of these lipid-based systems. These tools direct the methodical assessment of material properties and risk assessments during the formulation phase. This method guarantees that the finished product satisfies the appropriate requirements for efficacy, safety, and quality. The regulatory status and safety profile of nano lipid carriers are covered in the paper's conclusion, which emphasizes the importance of careful examination and oversight in bringing these cutting-edge products to market. Overall, this thorough analysis highlights the revolutionary potential of lipid-based nanocarriers and PEGylation in improving drug delivery and therapeutic efficacy, but it also draws attention to the continued difficulties and legal issues that need to be resolved in order to fully reap the benefits of these technologies in biomedicine.

## Introduction and background

Attaching polyethylene glycol (PEG) to nano lipid drug carriers prolongs their circulation time in the bloodstream by preventing recognition and removal by the immune system, particularly monocytes and macrophages. The PEG coating masks the carriers from opsonins, proteins that tag foreign particles for clearance by phagocytic cells, thereby allowing the drug to circulate for much longer durations compared to uncoated carriers [1]. Pharmaceutical companies use PEG with a molecular weight (MW) of over 20 kDa to create a "stealth" effect, reducing immune system recognition and extending the circulation time of drugs or nanoparticles (NPs). The thick hydration layer formed by long PEG chains minimizes protein adsorption and immune recognition, enhancing the stability, bioavailability, and therapeutic effectiveness of the medication. Using high MW PEG balances improved stealth properties with the management of the drug's physical and delivery characteristics [2].

The high MW PEG coating helps the drug carriers evade detection and clearance by the body's immune defenses, allowing the drug to circulate for an extended period [2]. The term PEGylation, denotes the strong chemical bond, a weaker interaction, or just sticking to the surface. It is frequently used to refer to PEG modification. PEGylation technology has been utilized to treat a variety of therapeutic modalities, such as aptamers, proteins, small molecules, and peptides [3]. PEGylation presents a number of difficulties in addition to its many advantages in medication delivery. Among these is the possibility of immunogenicity, since PEG can occasionally produce antibodies that could compromise the safety and efficacy of the treatment. PEGylation introduces another level of complexity to the production process, needing close supervision to guarantee stable PEG attachment and the therapeutic ingredient. Concerns about safety and regulations also arise from the possibility of PEG buildup, which makes continuous monitoring of the long-term effects of PEGylated medications in humans necessary. Last but not least, PEGylation can be expensive and scalable, which may have an influence on the general advancement and accessibility of these treatments [4]. As a result, there are currently over 30 PEGylated medications in use in clinical trials, in addition to numerous investigational PEGylated treatments [4]. A PEGylated enzyme replacement therapy (ERT) called Elfabrio (pegunigalsidase alfa-iwxj), which is used to treat Fabry disease, is one of the most recent successful FDA-approved medications. A stabilized recombinant variant of α-galactosidase A enzyme expressed in plant cells is called Elfabrio. It has undergone chemical modification [3,5].

Elfabrio, a PEGylated enzyme replacement treatment, has a half-life of 78.9 ± 10.3 hours and achieves stable pharmacokinetic features through chemical cross-linking with short PEG moieties. A different illustration is the PEGylated RNA aptamer avacincaptad pegol, which inhibits protein C5, blocking its conversion into C5a and C5b and lowering membrane attack complexes (MACs). Pegcetacoplan injection, or Syfovre, is a medication used to treat geographic atrophy (GA) and age-related macular degeneration (AMD) [6].

## Review

PEG characteristics

**Figure 1:**
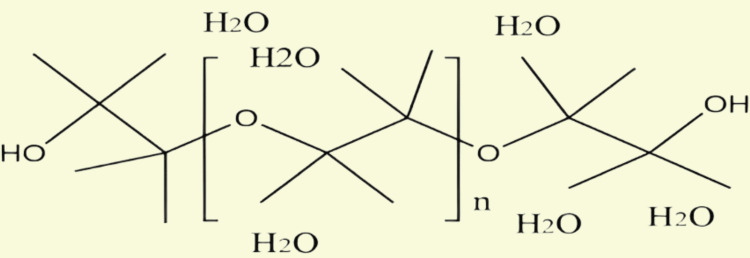
Structure of PEG to show its flexibility and hydration

**Figure 2:**
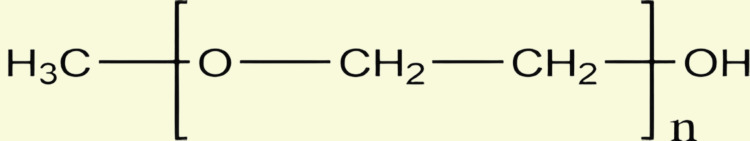
Structure of PEG to show its linear methoxy-PEG structure PEG, polyethylene glycol

**Figure 3:**
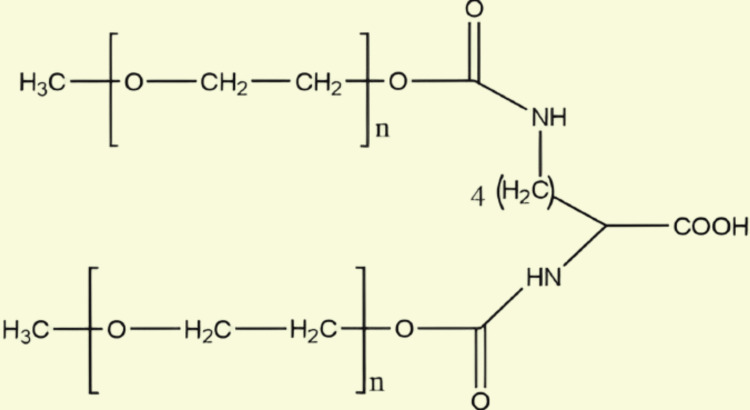
Structure of PEG to show its branched PEG structure PEG, polyethylene glycol

High geometric flexibility, biologic compatibility, amphiphilicity, not having steric barriers, and outstanding hydration capacity are just some of the widely recognized qualities of PEG. Water, ethanol, and many additional organic solvents, including dichloromethane, acetonitrile, acrylonitrile, and dimethylformamide (DMF), can all dissolve PEG [7]. The PEG is non-toxic and easily excreted from the body. PEGs with a MW of less than 30 kDa are typically excreted by the kidneys, while those greater than 20 kDa are primarily eliminated in the feces [6]. Furthermore, a variety of pharmaceutical applications can benefit from PEG because it is harmless and durable in the presence of acid, heat, and base. The properties of PEG products vary depending on their MWs. PEG with an average MW, which is between 200 and 700, is usually liquid at the surrounding temperature; MW > 700 causes a transition from partially solid to a soft solid (MW between 1,000 and 2,000) followed by hard crystal solid (MW > 2000). On the other hand, PEG's solubility in solvents made of organic matter, atmospheric pressure, liquid solubility, and freezing point all decrease as its molecular mass rises, but its viscosity, density by volume, boiling point, and freezing point all increase in parallel [4]. The structure of PEG showing flexibility and hydration is shown in Figure 1, its linear methoxy-PEG structure in Figure 2, and its branched PEG structure in Figure 3. The most commonly used activated PEG reactants are listed in Table 1.

**Table 1:** Most commonly used activated PEG reactants PEG, polyethylene glycol

S. No.	Selectivity	Linkage	Abbreviation	References
1.	Carbonyl			
	Amine	Amide	PEG-NH2	[5]
	Hydrazide	Amide	PEG-NNH2	[5]
2.	Amine			
	Acrylate	Amide	PEG-ACRL	[5]
	Tosylate	Amine	PEG-TS	[5]
3.	N-terminal			
	Aldehyde	Secondary amine	PEG-ALD	[5]
	Ortho-pyridyl thioester	Secondary amine	PEG-OPTE	[5]
4.	Thiol			
	Maleimide	Thioether	PEG-MAL	[5]
	Thiol	Disulfide	PEG-TS	[5]
5.	Disulfide bridge			
	Propenyl sulfone	Thioether	PEG-PS	[5]
6.	Miscellaneous			
	Isocyanate	Urethane	PEG-NCO	[5]
	Biotin	Non-covalent	PEG-BIO	[5]

Novel strategies of PEGylation

PEG has the ability to dramatically alter the surface characteristics of conjugated materials. Most of these materials have hydrophobic surfaces, which pose challenges for attaching PEG to them, thereby reducing the efficacy of PEG conjugation onto their surfaces. PEG is applied to the surface of NPs using three main strategies: covalent grafting to form a stable chemical bond, physical adsorption through electrostatic or hydrophobic interactions, and conjugation with hydrophobic molecules to create macromolecules that self-assemble with other compounds, resulting in PEGylated NPs in solution [5].

Novel Self-Assembly Approaches for PEGylated Nanocarrier Development

The hydrophilic PEG can interact with and form complexes with hydrophobic compounds, resulting in the formation of structures that possess both water-attracting and water-repelling characteristics. There are two primary methods used for the self-assembly of PEGylated nanocarriers: emulsification (also referred to as solvent evaporation or nanoemulsion) and nanoprecipitation (also known as solvent diffusion) [5,6]. Nanoprecipitation has been widely used to produce PEGylated nanocarriers for the targeted distribution of pharmaceutical medicines following intravenous (IV) administration. Since nanoprecipitation can be done in a single step and does not require significant energy inputs or substantial shear pressures, it is regarded as an environmentally conscious and low-energy technique for producing low-circulating nanocarriers. In this procedure, PEG-coupled moieties are dissolved with the drug(s) in a solvent mixture or an organic solvent that is soluble in water. In nanoprecipitation, the organic phase and the water component are gradually combined, either with or lacking surfactants [6].

As a result, during nanoprecipitation, the hydrophobic components separate from the water phase to form the central portion of the nanocarriers, while the hydrophilic PEG chains migrate to the NP surface in the aqueous phase. On the other hand, PEG-linked compounds or polymers are dispersed in an organic solvent or a combination of water-immiscible solvents throughout the emulsification process. The organic phase and the water phase are then gradually combined using emulsifying and either homogenization by high pressure or ultrasound. As the organic solvent evaporates in emulsion droplets, hydrophilic PEG chains tend to partition towards the oil/water interface. The pharmacological viability of the emulsification approach is determined by its ability to achieve higher loading capacities of drugs compared to the nanoprecipitation method [8].

Revolutionizing Nanocarrier Design with Physical Adsorption PEGylation

This method involves dissolving molecules containing PEG in an aqueous solution. Next, through hydrophobic, electrostatic in nature, or ligand-based connections, the PEG-containing molecules engage with the nanocarrier's surface. These interactions cause the PEG-containing molecules to physically adsorb or attach to the nanocarrier's surface, resulting in the PEGylation of the nanocarrier [7]. Pluronics are a type of triblock copolymer, specifically PEO-PPO-PEO (polyethylene oxide-b-polypropylene oxide-b-polyethylene oxide). Pluronics molecules are more easily adsorbed onto the hydrophobic surfaces of nanocarriers due to the hydrophobic bonds that exist between the PPO section and these surfaces. Furthermore, substances like vitamin E-d-α-tocopheryl-PEG 1000 succinate (TPGS)-PEG, lipid acid-PEG ester compounds, phospholipid-PEG, and other agents can be adsorbed onto polymeric and inorganic NPs to create a PEG corona. Coating nanocarriers with PEG-containing molecules that possess opposite charges can modify their surface charges. To regulate the negatively charged poly(lactic-co-glycolic acid) (PLGA) nanocarriers, for instance, PEG cation block polymers such as polyethylene glycol-polyethyleneimine (PEG-PEI) and polyethylene glycol-poly-l-lysine (PEG-PLL) can be bound. PEGylation facilitated the modification of core-shell NPs composed of PLGA and fusogenic peptide-liposome (FPL), resulting in NPs with optimized physicochemical properties suitable for the co-delivery of drugs and genes [9].

Numerous pharmaceutical benefits can be obtained from the efficient co-delivery of medications and genes using PLGA/FPL NPs, such as improved tumor-fighting efficacy, selective drug delivery to tumor tissues, improved drug internalizing it, conquering resistance to chemo longer circulating time, and increased absorption [7,9].

Chemical Conjugation-Driven PEGylation for Advanced Nanocarrier Design

**Figure 4:**
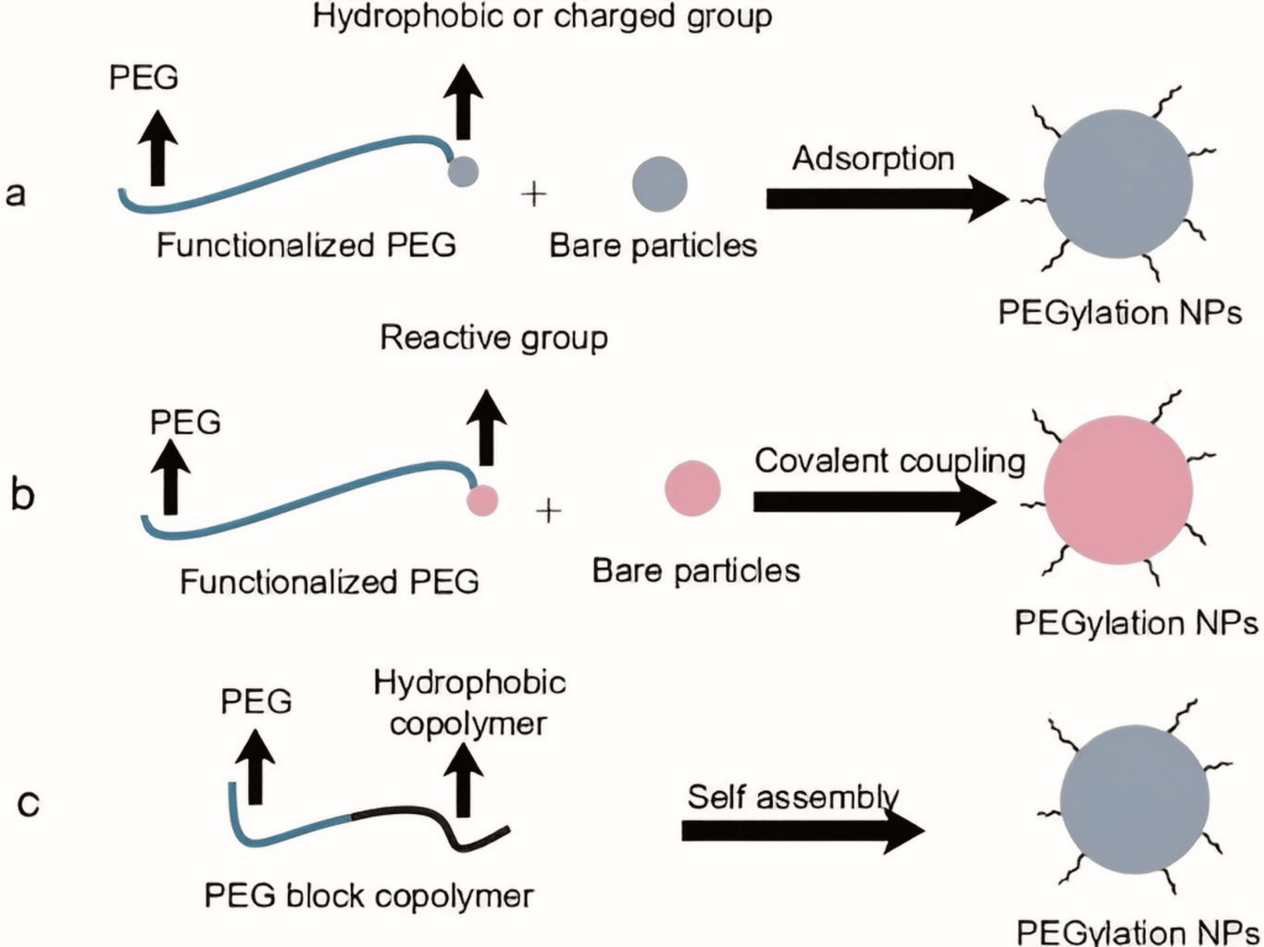
Novel strategies of PEGylation This figure is created by the author, Karthick Rajan S. PEG, polyethylene glycol; NPs, nanoparticles

PEG has been chemically conjugated to the surfaces of nanocarriers to accomplish PEGylation. PEG does not exist in the NP core because it is only present on the surface. As a result, products that have undergone PEGylation have a longer shelf life than those that have not. The shortcoming of this method is that the density and position of the surface-active site limit the grafting effects. Consequently, ensuring that the surface graft density meets application requirements is challenging. Nevertheless, batch-to-batch variations in the graft ratio can occur due to spatial hindrance and fluctuations in reaction rates. PEGylated polymeric nanocarriers were designed by Nance and associates to carry the anticancer medication paclitaxel, or PTX, to brain tissues. In comparison to uncoated polymeric nanocarriers, they found that PEGylation of nanocarriers significantly increases the trafficking of PTX to brain regions [1,2]. The novel strategies of PEGylation are shown in Figure 4.

PEGylation of nanocarriers reverses multi-drug resistance

MDR is one of the most difficult issues that cancer chemotherapy faces, among its many other challenges. Chemotherapy-resistant cancer tissues result in poor drug targeting, the protein P-g expression, respectively, and pumps called efflux that remove accumulated medicines from cells in the tumor. The main mechanism of drug resistance in cancer cells is the production of the membrane transporter P-glycoprotein (P-gp). Through this process, cancer cells become resistant to around 50% of anticancer medications, a phenomenon referred to as multidrug resistance (MDR) [10]. In recent decades, the creation of effective nanocarriers has emerged as a viable strategy to counteract MDR with chemotherapy. The created nanocarriers avoid P-gp-mediated drug resistance by internalizing without going through the efflux pump, either by stopping evacuation through direct contact with P-gp or by endocytosis. Recently, pH-responsive PEG-loaded docetaxel micelles of poly (acylhydrazone) were created to enhance tumor toxicity in vitro. Further modification of the micelles using glucose as the targeted ligand resulted in increased anticancer efficacy and decreased systemic toxicity poly [1]. In a different investigation, PEG phosphatidyl ethanolamine/vitamin micelles were created to co-deliver PTX and curcumin to patients with MDR human ovarian cancer. Both in vitro and in vivo, significantly greater toxicity was noted. PTX nanostructures activated with TPGS were used to fight MDR. TPGS functioned as a surfactant that served to stabilize the tiny crystals and as a P-gp inhibitor to reverse MDR [11]. It was discovered that PEGylated nanocrystals of PTX formulated with GPS (TPGS) were used to overcome MDR. In order to combat MDR, TPGS was used as a P-gp inhibitor as well as a surfactant that served to stabilize the NPs. Studies conducted in vitro and in vivo showed that PEGylated PTX nanocrystals markedly increased the antitumor activity in malignancies resistant to taxol patients [12].

PEGylation in biomedicine: challenges and effective countermeasures

The PEGylation technique has significantly improved selective internalization, accumulation, and plasma half-life, entering cancer cells, as well as a reduction in plasma clearance brought on by a decline in RES and MPS system detection and clearance. Despite these accomplishments, the widespread adoption of PEGylation in the development of anticancer nanomedicines faces several challenges and limitations. PEGylation is the process of joining PEG molecules to pharmaceuticals to prolong their plasma half-life. Due to this alteration, the drug is larger and more hydrophilic, which slows down the rate at which enzymes break it down and lessens the kidneys' ability to remove it from the bloodstream. As a result, PEGylated medications are more effective and require fewer doses since they are in circulation for longer. PEGylation improves drug penetration into cancer cells via leaky vasculature through the enhanced permeability and retention (EPR) effect, and it extends plasma half-life by shielding drugs from recognition and subsequent removal by the mononuclear phagocytes system (MPS) and the reticuloendothelial (RE) unit. However, studies show that the hydrophilic outer layer (PEG coating) frequently prevents PEGylated nanocarriers from engaging with biological tissues and malignant cells, which results in insufficient cell uptake and decreased anticancer activity [8]. This problem can be more challenging in actively directed nanocarriers when ligand-receptor interaction is necessary to provide therapeutic benefits. The PEG's existence on nanocarrier surfaces inhibits the interaction between surface-grafted ligands and biological targets (such as receptors or biomarkers) that are overexpressed on tumor cells or tissue surfaces. To tackle these challenges, recent designs of nanocarriers have been developed with a strategy centered on microenvironment-triggered cleavage of PEG from the nanocarrier's surface. The list of FDA-approved PEGylated drugs is listed in Table 2.

**Table 2:** List of PEGylated drugs approved by the FDA GCSF, granulocyte colony-stimulating factor; GLA, galactosidase alpha; MW, molecular weight; PEGs, polyethylene glycol

Trade Name	Company	PEGylated Entity	Indications	Average MW of PEGs	Approved
Macromolecular drugs					
Elfabrio (pegunigalsidase alfa-iwxj)	Chiesi Global Rare Diseases/Protalx	Recombinant human GLA enzyme	Fabry disease	~2 kDa	2023
Izervay (avacincaptad pegol)	Iveric Bio	Ribonucleic acid aptamer	Geographic atrophy	~43 kDa	2023
Syfovre (pegcetacoplan injection)	Apellis	Pentadecapeptide	Geographic atrophy	40 kDa	2023
Rolvedon (eflapegrastim-xnst)	Spectrum Pharmaceuticals	G-CSF	Febrile neutropenia	3.4 kDa	2022
Stimufend (pegfilgrastim-fpgk)	Fresenius Kabi	G-CSF	Neutropenia	20 kDa	2022
Fylnetra (pegfilgrastim-pbbk)	Amneal Pharmaceuticals LLC	G-CSF	Neutropenia	20 kDa	2022
Besremi (ropeginterferon alfa-2b)	PharmaEssentia Corp	Interferon	Polycythemia vera	40 kDa	2021
Skytrofa (lonapegsomatropin)	Ascendis	Human growth hormone	Growth hormone deficiency	4 × 10 kDa	2021
Empaveli (pegcetacoplan)	Apellis	Pentadecapeptide	Paroxysmal nocturnal hemoglobinuria	40 kDa	2021
Nyvepria (pegfilgrastim-apgf)	Pfizer Inc.	G-CSF	Neutropenia associated with chemotherapy	20 kDa	2020
Esperoct (turoctocog alfa pegol)	Novo Nordisk	Recombinant antihemophilic factor	Hemophilia A	40 kDa	2019
Ziextenzo (pegfilgrastim-bmez)	Sandoz	G-CSF	Infection during chemotherapy	20 kDa	2019
Small molecular drugs					
Movantik (naloxegol)	AstraZeneca	Naloxone	Constipation	339 Da	2014
Asclera (polidocanol)	Chemische Fabrik Kreussler & Co.	Dodecanol	Varicose veins	4,000 Da	2010
Nanoparticles					
Spikevax	Moderna	Lipid nanoparticles	Prevention of COVID-19	2 kDa	2020
Comirnaty	BioNTech/Pfizer	Lipid nanoparticles	Prevention of COVID-19	2 kDa	2020
Onpattro (patisiran)	Alnylam Pharmaceuticals	Lipid nanoparticles	Polyneuropathy of hereditary transthyretin-mediated amyloidosis	2 kDa	2018

Examples of PEGylated alternatives are listed in Table 3.

**Table 3:** Examples of PEGylated alternatives Ala, alanine; Asp, aspartic; Gly, glycine; Pro, proline; Thr, threonine; Ser, serine; Fc - fragment crystallizable

Technology	Material	Type	Applications	Status	References
PASylation	Pro, Ala, and Ser polypeptide	Disordered polypeptide	Proteins	Pre-clinical	[2]
PMeOx	Poly(2-oxozoline)	Amphipathic polymer	Proteins, liposomes, nanomaterials.	Pre-clinical, early clinical	[2]
PCB	Poly(carboxybetaine)	Zwitterionic polymer	Proteins, liposomes, nanomaterials.	Pre-clinical	[2]
XTEN	Ala, Asp, Gly, Pro, Ser, and Thr polypeptide	Unstructured polypeptide	Proteins	Early clinical	[2]
Fc fusion	Immunoglobulin Fc domain	Protein domain	Proteins	Clinical approved	[2]

Safety profile of PEG

PEG has extensive applications in numerous medicinal compositions. They are typically thought of as non-irritating and non-toxic compounds. However, PEG has been linked to adverse effects; low MW glycols are the most hazardous. These include modified pharmacokinetics, which might result in unanticipated drug distribution; immunogenicity, where anti-PEG antibodies cause allergic reactions and decrease medication efficacy; and potential hepatic and renal toxicity from the buildup of PEGylated substances. Modifying PEG's size or structure, utilizing different polymers, desensitizing patients, picking PEGylation sites carefully, and closely monitoring patients for negative reactions are all examples of mitigation techniques. These strategies seek to minimize the hazards associated with PEGylation while balancing its advantages [8].

Nevertheless, glycols are not very poisonous. Topically applied PEGs have the potential to cause harm, particularly when applied to mucosal membranes. There have also been reports of delayed allergic responses and urticaria caused by the topical use of PEGs [10,12]. When PEGs are applied topically to burn victims, the most dangerous side effects are hyperosmolarity, metabolic acidosis, and renal failure. Patients with severe burns, open wounds, or renal insufficiency should thus use topical treatments containing PEGs with caution. Large doses of orally administered PEGs can induce a laxative effect. In therapeutic settings, patients undergoing colon cleansing may be given up to four liters of an aqua composite containing high MW PEGylated polymer and electrolytes. When orally consumed, liquid PEGs can be absorbed to some extent, but higher MW PEGs are generally not significantly absorbed in the gastrointestinal tract. Low MW PEGs may undergo partial metabolism, but when PEG integration is primarily excreted as an intact compound in urine. The World Health Organization (WHO) recommends a daily intake of PEGs of up to 10 mg per kilogram of body weight. In parenteral preparations, it is recommended to limit the PEG 300 proportion to about 30% v/v, as concentrations higher than around 40% v/v have been associated with hemolytic effects [8].

Regulatory status of PEG

PEG, recognized as generally recognized as safe (GRAS) by the FDA and with a long-established safety record in humans, is the industry standard “stealth” polymer for drug delivery. PEG is utilized in oral, topical, and IV preparations, with its maximum dosage specified in the US FDA's Inactive Ingredient Guide (IIG), also known as the Inactive Ingredient Database (IID). PEGylating proteins have been used in medicine to elude the immune system, lengthen circulation lives, and reduce immunogenic potential - the capacity of a non-self-substance to elicit a body's immune activity. Similarly, PEG coating, commonly referred to as "PEGylation," is widely used as a strategy to enhance the efficacy of the conveyance of drugs and genes to desired cells and tissues [8,12].

Doxil®, the revolutionary PEGylated NP (NP) debut, was approved by the FDA in 1995. One week after injection, the bioavailability of the anticancer drug doxorubicin in Doxil "Stealth®" liposomes was more than 90 times higher than that of the non-conjugated drug, with a 72-hour drug half-life and a 36-hour circulation half-life. Investigations suggest that to guard NP surfaces from protein attachment and lower detection by the mononuclear phagocyte system (MPS), a PEG MW of 2 kDa or higher is imperative [7].

PEG is the primary ingredient in anti-freeze formulations. PEG is utilized in drug administration systems due to its water solubility, neutrality, lack of toxicity, and ability to form biocompatible and biodegradable micelles. As an anti-deposition agent, PEG is beneficial in detergent compositions. PEG serves as a robust anti-redeposition agent for polyester textiles, synthetic-cellulose apparel, and polyester-cotton blends. Powder formulations employ PEG as a binder to effectively guarantee that all of the constituents are retained together as granules. The formulation's decision on the amount of high- or low-melting PEG is typically made in light of the local temperature in the powder detergent product's intended distribution or sale [6].

In addition to the functions and applications for glycol that have already been discussed, PEG is non-corrosive due to its non-toxic nature. Dental preparations, intramuscular and IV injections, ophthalmic formulations, oral capsules, solutions, syrups, and tablets, along with topical, vaginal, and rectal formulations, are categorized as non-parenteral medications licensed in the UK. In Canada, PEG is listed as an acceptable non-medicinal ingredient [12].

Classes of lipid-based nanocarriers

**Figure 5:**
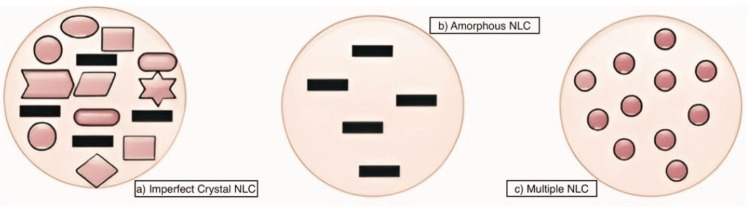
Varieties of nanostructured lipid carrier (NLC) based on lipid and oil composition differences and fabrication techniques This figure is created by the author, Karthick Rajan S.

Various classifications of nano lipid carriers can be delineated according to their physicochemical characteristics and manufacturing methods. Different categories of nano lipid carriers can be identified based on their physical and chemical properties as well as how they are produced [13]. Phospholipid bilayers enveloping aqueous cores define liposomes, which are spherical vesicles composed of cholesterol and other naturally occurring phospholipids. Numerous liposome formulations are currently approved for treating a wide range of conditions. Among the drugs encapsulated in liposomes are Amphotericin B (Ambisome®) for severe fungal infections, cytarabine (Depocyt®) for lymphomatous meningitis, doxorubicin (Doxil®) for treating different types of cancers, Myocet®, and Lipodox® for cancer, DepoDur® utilizes morphine sulfate for effective pain management, Epaxal® employs inactivated hepatitis A virus to prevent hepatitis A, and Inflexal® utilizes inactivated hemagglutinin from influenza virus strains A and B for influenza protection, showcasing their diverse applications in medicine. RE system clearance of liposomes occurs quickly, and they are unstable and have a short shelf life when it comes to encapsulating hydrophilic medicines [5]. Niosomes are lipid-based vesicles containing non-ionic surfactants and cholesterol or its derivatives. They are characterized by improved environmental compatibility and physical durability compared to liposomes, and they can load both hydrophilic and lipophilic compounds. Emulsifying agents help stabilize solid lipids in an aqueous dispersion, which is what makes up solid lipid nanoparticles (SLNs). SLNs are capable of efficiently delivering a wide variety of compounds, encompassing both lipid-soluble and water-soluble medications - vaccinations, DNA fragments, genetic material, protein fragments, and amino acid chains. They also offer the advantages of being biocompatible, producing goods quickly and efficiently, being scalable in the manufacturing process, and, in many situations, not requiring the use of organic solvents in the process. Lipids' crystalline structure reduces their ability to load drugs. The medication is expelled as a result of lipids crystallizing into an ordered lattice (β-modification). Moreover, gelation may happen during storage in the dispersion phase. To tackle the drawbacks associated with SLNs, researchers developed a newer generation of SLNs called nanostructured lipid carriers (NLCs). At room temperature as well as physiological body temperature, NLCs preserve the integrity of their solid lipid matrix. However, NLCs incorporate an oil phase alongside the solid lipid, creating a less organized lipid matrix. This modification enhances drug loading capacity and prevents drug leakage during storage [14]. Varieties of NLC based on lipid and oil composition differences and fabrication techniques are shown in Figure 5.

Imperfect NLC refers to the idea of mixing lipids of different structural compositions, like glycerides made up of many fatty acids, which causes the crystalline structure to be disrupted. Drug loading can be further improved by using a combination of glycerides with varying degrees of saturation and carbon chain lengths, thereby increasing imperfections in the lipid matrix. In the amorphous kind, the solid lipid is combined with specific lipids like isopropyl myristate or hydroxyoctacosanyl hydroxystearate to generate a structureless amorphous matrix. Consequently, NLCs maintain the drug in an amorphous form instead of an ordered one, preventing drug ejection caused by β-modification during storage. There are multiple O/F/W type NLC formulations with distributed nanosized oil-like compartments in a dense matrix. These nanoscale compartments have better drug solubility, which leads to higher drug loading. Furthermore, a thick lipid matrix around the compartments prolongs the release [15].

Exploring lipid-based formulations: characterization and development

A critical part of product development is thorough characterization, which encompasses analyzing sterility, pH levels, lipid content, particle or droplet size, encapsulation or drug loading efficiency, and residual solvent content. Other essential parameters to consider include viscosity, rheology, osmolality, related substances, heavy metals, and the elemental profile. The profile of extractables and leachables (E&L) in the formulation has become a mandatory regulatory requirement in the development of complex products. Particle dimensions and potential zeta measurements can be used to evaluate physical stability. The polydispersity index (PDI) and particle size variability analysis equipment are utilized to determine the SPAN value, calculated as (d90 - d10) / d50). Dynamic light scattering (DLS) and laser light scattering are techniques used to evaluate zeta potential. The pharmaceutical industry is increasingly focused on target-site drug delivery systems because of their benefits, including improved bioavailability and greater drug dosage capacity [5]. However, there are certain issues that must be addressed, such as the interaction between drug delivery devices and blood proteins. One promising approach to tackle this issue involves surface modifications using PEG. This method enhances the solubility of the original drug, prolongs its presence in the bloodstream, reduces the likelihood of provoking an immune response, and minimizes adverse effects. PEG can be attached to peptides, proteins, and NPs either covalently or non-covalently, decreasing their immunogenicity, facilitating blood-brain barrier crossing, and limiting RE system clearance. PEGylation has been successfully utilized across various therapeutic fields, including small molecules, aptamers, peptides, and proteins, resulting in over 30 PEGylated medications currently in use and numerous experimental PEGylated compounds undergoing clinical assessment. The potential of PEGylation to drive innovation and progress in therapeutic agent development is significant, especially given the rapid growth of macromolecular drugs and the emergence of novel treatments like immunotherapies. This suggests that PEGylation is poised to significantly impact the future of drug development, particularly in expanding avenues for macromolecular medications and advancing innovative immunotherapeutic strategies [16].

**Figure 6:**
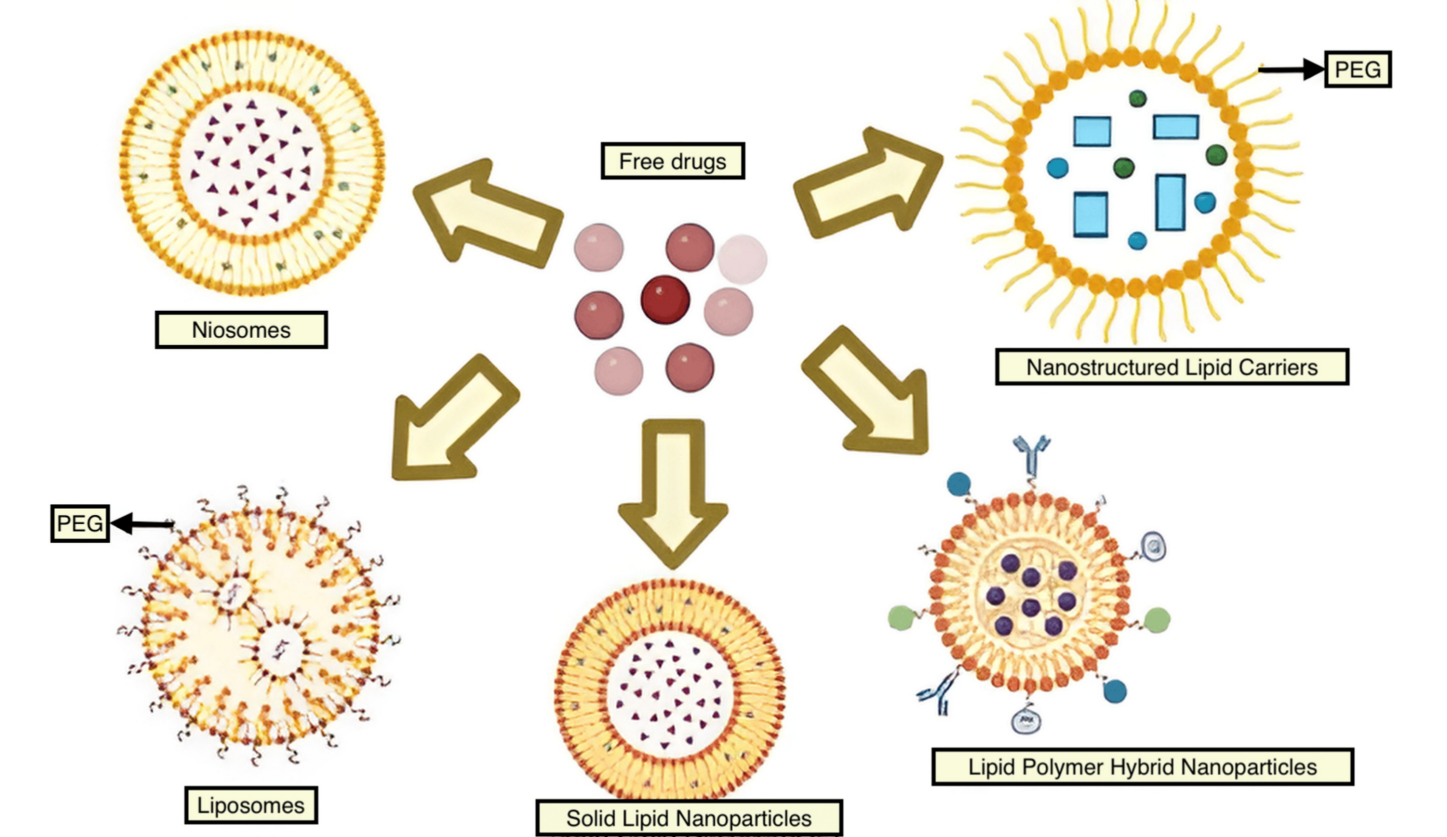
PEGylation coating on various nanocarriers This figure is created by the author, Karthick Rajan S. PEG, polyethylene glycol

The methods of atomic force microscopy and electron microscopy can be used to measure surface topography and morphology. Lipid/excipients ratio and lipid contents are particularly important to identify in lipid-based formulations and can be analyzed by powder X-ray diffraction (PXRD), gel permeation chromatography, high-performance liquid chromatography (HPLC), etc. To investigate polymorphic transformations or crystallinity in lipid excipients. It is essential to determine the importance of both in vivo research and in vitro studies in evaluating the permeability of oral and topical formulations. Advanced thermal analysis techniques such as thermogravimetric analysis (TGA) and differential thermal analysis (differential scanning calorimetry (DSC)) are utilized for evaluating the thermal behaviors of lipids, excipients, polymers, and active pharmaceutical ingredients (APIs). According to the International Council for Harmonisation of Technical Requirements of Pharmaceuticals for Human Use (ICH) guidelines, assessing the stability of lipid-based products is crucial for determining shelf-life, degradation patterns, and expiration dates under specified storage conditions, which must be clearly stated on the product's label or leaflet. Furthermore, in order to pack the product, the different key evaluation parameters of the container-closure system must be carried out in accordance with regulatory standards [15]. PEGylation coating on various nanocarriers is shown in Figure 6. The core ingredients driving NLC technology are listed in Table 4.

**Table 4:** Core ingredients driving nanostructured lipid carriers (NLC) technology

Formulation Ingredients	Examples	References
Solid lipids	Glyceryl palmitostearate	[7]
	Carnauba wax	[17]
	Stearic acid	[17]
	Glyceryl behenate	[7]
	Cetyl palmitate	[13]
	Grade of Witepsol	[7]
	Grades of Softisan	[7]
Liquid lipids	Soybean oil	[13]
	Medium-chain triglycerides/caprylic and capric triglycerides	[7]
	Oleic acid	[7]
	Isopropyl myristate	[17]
	α-Tocopherol/vitamin E	[17]
	Corn oil	[13]
	Squalene	[7]

Methods of NLC preparation

There are numerous kinds of protocols available for NLC preparation. The method of high-pressure homogenization, utilizing elevated temperature and pressure concurrently, stands as the most extensively employed approach. The homogenization approach using high pressure and low temperature is another. The methodologies used to prepare SLNs can also be applied to prepare NLCs. In addition to solvent dispersion, these techniques also include elevated temperature emulsion formation followed by cold curing, high-pressure homogenization, ultrasonic emulsion evaporation, membrane contactor, microchannel, and microtube methods [18]. Nano lipid carrier preparations are listed in Table 5.

**Table 5:** Nano lipid carriers preparations

Technique	Process	Benefits	Drawbacks
High-intensity homogenization (HPH)	In the hot, high-pressure homogenization (HPH) method, a bioactive lipid melt is homogenized with an aqueous stabilizing agent solution kept at the high-pressure thermal processing, generating a heated pre-emulsion with strong shear. Subsequently, this pre-emulsion is exposed to HPH and heated above the lipid melting point. After cooling to ambient temperature, the resultant nano-emulsion recrystallizes to generate nanostructured lipid carriers (NLCs).	Simple and cost-effective.	Temperature elevation during homogenization is inevitable.
a. Thermal homogenization	A drug-lipid melt is first consolidated and then quickly ground under liquid nitrogen in cold, high-pressure homogenization, producing microparticles. Following being dispersed in a cool surfactants solution, these microparticles are exposed to the HPH at ambient temperature or less.	Well-established at a large scale.	The quality of dispersion is frequently affected by the presence of microparticles.
b. Low-temperature homogenization	For the formation of nanoemulsions, three to five homogenization cycles at pressures between 500 and 1,500 bar are usually sufficient.	Organic solvent-free manufacturing is preferred. Drugs that are thermolabile can be handled under cool conditions.	-
Microemulsion	At the same temperature, a hot aqueous surfactant solution is added to the drug-lipid melt to create an emulsion. After a short while, the hot microemulsion is rapidly added to cold water, creating a nanoemulsion that recrystallizes to create NLCs.	Scale up feasible.	Uses enormous amounts of water and high surfactant concentrations, which significantly dilutes the particles.
Solvent diffusion method	Benzyl alcohol, respectively, are examples of organic solvents that are used to dissolve the lipids. These organic solvents are saturated with water to achieve thermodynamic equilibrium. The dispersed phase solidifies when the transitory oil-in-water emulsion is subsequently gradually added to water while being constantly stirred.	Water-immiscible solvents are employed.	Use of organic solvents.
Emulsion solvent evaporation technique	The lipids are dissolved using organic solvents that are water-soluble such as trichloromethane. After that, the fatty acid solution is continuously stirred while being emulsion in an aqua surfactant solution. The non-polar solvents are then evaporated, leading to lipid precipitation.	Prevents thermal stress, making it appropriate for medications sensitive to heat.	Ultrafiltration or lyophilization requires the use of solvents that are organic.
Emulsification sonication method	The procedure is similar to HPH, but in this case, the pre-emulsion undergoes ultrasonication using a probe sonicator.	Intense mixing under high shear conditions.	An additional evaporation step is required, and there may be concerns about metallic contamination from the sonication probe, making the process more complex and potentially tedious.
Phase transition method	Lipids, medication, liquid, and surfactants are combined, heated, and cooled three times, reaching a temperature of 85-60-85°C. Subsequently, a shock is induced by rapid dilution with cooled water (0°C), resulting in the formation of NLCs through phase inversion.	Suitable for thermosensitive drugs. Avoids use of organic solvents.	Tedious process.
Solvent displacement method/solvent injection	By using an injection needle and a water-dispersible solvent, the lipids are quickly dispersed into an aqueous surfactant solution. This process, known as the displacement method, makes it simple to handle and quickly uses organic solvents.	A production process that is easy to handle and allows for fast implementation.	Use of organic solvents
Contractor membrane technique	Little lipid droplets are created when the lipid melt is forced across a permeable barrier. Lipid droplets are swept out of the membrane's modules by the concurrent circulation of the aqueous phase. Once cooled to room temperature, lipid nanoparticles, or NPs, are created.	A straightforward approach with minimal equipment requirements.	-

Research and development of nanocarriers

Colloidal NPs, known as nanocarriers, are mostly employed to deliver medications or other compounds to certain target locations. The diameter of nanocarriers usually varies from one to hundred nm. Nevertheless, in order to be used for medicinal purposes, nanocarriers need to be smaller than 200 nm in order to pass through the human body's microcapillaries, which are likewise a wavelength of approximately 200 in size. Because these nanocarriers are typically thought to be harmless and inactive, they offer good biocompatibility [13]. Due to the drug's continuous release circumventing the endosome-lysosome pathway, these nanocarriers will have a prolonged circulation time. It is possible to maximize the effectiveness of nanocarriers while reducing their adverse consequences by altering their physicochemical features, such as their chemistry, design, and coating qualities. Consequently, this significantly influences drug delivery. Despite the wide array of nanocarriers developed, only a few demonstrate exceptional capability in effectively delivering medications to their intended targets. Among the distinctive characteristics of nanocarriers are the following: sustained and targeted drug delivery, reduction in toxicity, enhanced stability, enhanced solubility, enhanced biodistribution, and pharmacokinetics [17].

Organic nanocarriers

Organic nanocarriers include lipid-core NPs, lipid bilayer carriers, Dendritic macromolecules, polymer nanosphere, self-assembled nanocarriers, and engineered viruses. Organic nanocarriers offer remarkable versatility, are minimally toxic, and are capable of conjugating a wide variety of drugs and ligands for effective therapeutic delivery. Micelles and liposomes, both types of organic nanocarriers, can accumulate at target sites due to their EPR effects. The first generation of nanocarriers includes polymeric nanocarriers and liposome-mediated drug delivery because they have a simple excipient [7].

SLNs

Solid NPs of lipids have been widely used as efficient lipophilic drug delivery methods. Melted solid lipids are dissolved in water to create SLNs, and their stability is enhanced by incorporating emulsifiers through high-pressure homogenization or micro-emulsification techniques. Solid lipids at room temperature, such as unbound fatty acids, hydroxyl compounds, lipid esters, monoacylglycerols, glycerol diesters, and triacylglycerols, are commonly used to create solid lipid nanocarriers. The solid lipid's framework, body, or interior can include therapeutic molecules, depending on the production conditions and content. This solid lipid nanocarrier's adaptability allows it to overcome the limitations of conventional treatments. The RE system can efficiently clear conventional solid lipid nanocarriers, posing a challenge for sustained drug release, especially when combining hydrophilic and ionic drug molecules [19]. Recently, solid lipid nanocarriers have been explored not only for incorporating lipophilic anticancer drugs but also for integrating ionic and hydrophilic pharmaceuticals. For example, oral medication delivery using polymer-lipid hybrid small carriers has been investigated as a potential strategy. Recent findings indicate that newer nanocarrier technologies can mitigate the limitations of conventional solid lipid nanocarriers. These advancements include lipid drug conjugates, which are carrier molecules insoluble in water, and lipid-based NPs, containing mixtures of oils and solid fats. Drug delivery with this nanocarrier can occur orally, parenterally, or through topical application. The ideal tailor-made carrier for delivering any medicinal molecule to a designated place is the solid lipid nanocarrier [20]. Solid lipid nanocarrier research has been done for a number of purposes, including targeted drug delivery of antitumor agents, controlled release of active agents, treatment of ophthalmic diseases, and delivery of genes and nucleic acids [14,19].

*Nano Liposomes * 

Both lipophilic and hydrophilic medications can be targeted to specific sites using liposomes, which are spherical lipid vesicles composed of double-layered lipid membranes surrounding a hydrophilic core. Lipid NPs can be categorized based on their structure: They can either be uni-lamellar vesicles, consisting of a single lipid bilayer, or multilamellar vesicles, containing multiple lipid bilayers. These vesicles serve as carriers for biologically active chemicals targeted to specific locations. However, these compounds typically have shorter half-lives in systemic circulation [14].

Thus, liposomes can be coated with polymeric molecules such as PEG to create PEGylated liposomes, also known as concealment liposomes. Because of its ability to evade clearance by the RE system, this stealth liposome enables sustained drug release and possesses a prolonged half-life in the bloodstream. The therapeutic molecules that are integrated into the liposomes enhance their pharmacokinetics and biodistribution. For example, doxorubicin in a stealth liposome is less likely than the drug in solution to decrease drug distribution in plasma and concentration in healthy cells. According to reports, temperature response liposomes and other controlled switch nanocarriers can improve local medication release [17].

Nano Dendrimers

According to dendrimers, they are branched macromolecules with an initiator core at the center that gives birth to many arms or terminal active groups. Amino acids, sugar compounds, and nucleotides can all be converted into dendrimers. They are a special source for drug delivery because they are multivalent, extensively branched, have a variety of peripheral groups, and have a distinctive MW. An uneven, well-organized dendrimer branching pattern can result from the sequential synthesis of dendrimers. A generation starts with an initial core introduction, followed by external group expansion. Drug molecules can be housed within core voids through improved surface functionality involving hydrogen bonds, hydrophobic interactions, or chemical bonding [11]. Covalent bonds can also bind drug molecules to terminal active groups. Dendrimers have a clearly defined structure that may be carefully customized to encapsulate different medications, including rifampicin, an anti-tuberculosis treatment. Single-generation dendrimers have the potential to cause the molecules they are connected to dissociate. The creation of chemical and physical bonds is the main method via which drugs interact with dendrimers. This dendrimer has applications in gene delivery, medication delivery, antiviral drug delivery, and magnetic resonance imaging scanning. It is also proven to be helpful when connected to prodrugs. The dendrimer is closely associated with several anticancer medications, including doxorubicin and cisplatin, to provide increased anticancer activity [11,19].

Polymeric Nanocarriers

Polymeric nanocarriers are colloidal, solid NPs made of any biodegradable polymer, which are known as polymeric nanocarriers. It can either be a matrix type (nanospheres), where therapeutic molecules are trapped inside the polymer matrix, or a reservoir type (nanocapsules), where drug molecules are dissolved or dispersed within the polymer core. Additionally, the medicine can chemically bond to or adsorb on the surface of both types. When polymeric nanocarriers biodegrade in the human body, they release monomers that are quickly broken down by the processes of metabolism. Both organic and synthetic polymers are utilized to fabricate polymeric nanocarriers. Natural polymers encompass chitosan, gelatin, albumin, collagen, and alginate. Compared to alternative nanocarriers, polymeric nanocarriers offer advantages in terms of durability, drug encapsulation efficiency, plasma half-life, and sustained drug release [14]. To target malignant cells, an anticancer medication, such as doxorubicin, is encapsulated within polymeric nanocarriers. The physicochemical properties of the polymeric material can be modified to enhance controlled drug release. Moreover, multifunctional polymeric nanocarriers capable of incorporating multiple drugs can be developed. Additionally, advances are being made in the synthesis of polymeric NPs for targeted drug administration using trigger-responsive polymers. These smart polymers release the drug in response to both exterior and interior stimuli, such as thermal stimulus, photonic stimulus, acoustic stimulus, magnetomotive stimulus, and electromotive stimulus that cause the release of the medication, including acidic conditions, oxidation-reduction reactions, biocatalysts. The design of smart polymers has several obstacles, including as scalability, biocompatibility/toxicity, and stimulus sensitivity. Extrinsic stimuli, on the other hand, present difficulties such as those relating to tissue penetration, localization, and compliance, whereas intrinsic stimuli face variations between clinical and preclinical models. A polymeric nanocarrier is potentially useful for targeted drug delivery, notwithstanding these difficulties [14].

Micelles

The term "micelle" was introduced by Micelles McBain in 1913 to describe the colloidal aggregates formed when detergent is mixed with water, creating a liquid colloid. These molecules are amphiphilic, with a polar head that is hydrophilic and in touch with the external solvent and a nonpolar tail that is hydrophobic and facing the center. Similarly, an amphiphilic or detergent molecule in a nonpolar solvent can create an inverse micelle, with its hydrophilic head oriented towards the center and its hydrophobic tail positioned outward. The parameters of the amphiphilic molecule and the solution, including temperature, ionic strength, and pH, affect the dimensions and geometry of the micellar NPs that are produced. Micelle formation is governed by the critical micellar concentration, which refers to the concentration of surfactant required for micelle formation. Proper micelle production will not happen below the critical concentration of micellar particles [11]. Two copolymers are used to create polymeric micelles in specific solvents in addition to amphiphilic molecules. In a solvent, one copolymer dissolves while the other is insoluble. In order for the copolymer to create the chain or micellar, the soluble copolymer produces the shell, and the insoluble copolymer forms the core. This polymeric micelle presents itself for use in pharmaceutical and industrial settings.

These advanced polymeric micellar nanocarriers effectively target the pilosebaceous unit, offering valuable therapeutic benefits for conditions affecting hair follicles. For instance, the targeting efficacy of adapalene, when encapsulated in these micellar nanocarriers, increased significantly to 4.5-fold, demonstrating a 3.3-fold enhancement with a specific dosage. In the realm of oligonucleotide delivery, a micellar nanocarrier responsive to pH and containing a fluorescently labeled aptamer directed against the human epidermal growth factor receptor exhibits potential for efficiently transporting nucleic acids to targeted cancer sites [14].

Inorganic nanocarriers

Inorganic nanocarriers with stability and functional flexibility, such as gold and silica NPs, are useful for drug delivery. However, because of their capacity to pass through biological barriers and accumulate in organs, they provide health hazards, including toxicity and immunological reactions. Their continued presence in ecosystems raises environmental issues since they may endanger wildlife and contaminate soil and water. These hazards draw attention to the necessity of a thorough life cycle assessment, safer design techniques, and stricter laws to guarantee their safe use and disposal. Mesoporous silica, magnetic nanocarriers, quantum dots, gold, and other materials are examples of inorganic nanocarriers. Mesoporous silica nanoparticles (MSNs) exhibit cytotoxicity, which can result in oxidative stress and cell damage. Additionally, they are less biodegradable, which may cause inflammation and a long-term buildup in tissues. Although silicic acid, which is usually regarded as harmless, is released when MSNs degrade, partial disintegration may result in persistent toxicity. In order to address these problems, surface changes that improve biocompatibility and degradation must be used in conjunction with meticulous dosage management and extensive toxicity studies. The tractable features are exploited by the inorganic nanocarriers. Effective applications for inorganic nanocarriers include biosensing, cell labeling, targeting, imaging, and diagnosis. These inorganic nanocarriers also have synergistic therapeutic effects. Furthermore, exceptional magnetic, plasmonic, and optical properties can be enabled by modifying the size or composition of the inorganic nanocarriers. On the other hand, using toxic metals as inorganic nanocarriers may eventually give rise to long-term health issues [17]. Because hazardous metals have the ability to leak out of the body and accumulate there, using them with MSNs can have long-term health consequences. Prolonged exposure can lead to nephrotoxicity, which harms the kidneys, and neurotoxicity, which impairs brain function. Furthermore, there is a higher chance of carcinogenic effects, where prolonged exposure to metals may hasten the onset of cancer. These dangers emphasize how crucial it is to reduce the amount of hazardous metals used in nanocarrier systems and make sure thorough safety evaluations [17].

Carbon Nanotubes

Because of their unique biological and physical characteristics, carbon nanotubes are a perfect and exciting source for medication delivery. These structures consist of hollow tubes where graphene sheets are precisely wrapped together at specific angles. The quantity of sheets of graphene wrapped around one another determines the number of walls in the carbon nanotube structure, which can be mono or multi-walled. These tubes have diameters ranging from 0.4 to 100 nm and lengths that can extend thousands of times their diameter. Carbon nanotubes are commonly used in drug delivery procedures due to their remarkable qualities, which include a large aspect ratio, extremely low weight and a huge surface area, needle-like nanoscale shape, and unique biological, thermal, mechanical in nature, and electromagnetic capabilities. Due to its needle-like penetration capability, carbon nanotubes can easily facilitate the endocytosis process by penetrating obstacles or cell membranes. The functionalized nanotubes have a prolonged half-life in the serum and are soluble in water [11]. Non-functionalized carbon nanotubes, on the other hand, are poisonous and insoluble in water. Its flexibility, surface modification, and structural stability make it an effective agent for targeting cancer cells. According to that theory, targeted drug administration is achieved by encapsulating or coupling anticancer medications such as methotrexate, PTX, mitomycin C, doxorubicin, and others with functionalized carbon nanotubes. Because of their inherent qualities, carbon nanotubes are a perfect source for numerous industrial applications in addition to their use in biomedicine. Another significant carbon-based nanocarrier that works well in medicine is graphene delivery [14].

Gold Nanocarriers

Nanocarriers composed of gold, a noble metal, have revolutionized nanotechnology by enabling the creation of gold NPs. These NPs are utilized effectively in various applications such as gene therapy, photothermal therapy, surface-enhanced resonance spectroscopy, chemotherapy, and photoacoustic imaging. Gold NP synthesis can be accomplished by top-down or bottom-up methods. Gold NPs exist in various anisotropic forms, such as nanostars, nanorods, nanocages, nanoshells, and nanoprisms. The primary characteristic of gold nanocarriers that draws them to the biomedical field is their optical property [11]. It makes it possible for many biomolecules to bind to the gold nanocomposites, including biological catalysts, saccharides, oligopeptides, biological macromolecules, and genetic code. This makes it possible for molecules to move through the cell's barriers and inside it effectively. The primary use of gold nanocarriers is to efficiently image tumor cells. Potential three-dimensional pictures of tissues can be obtained using the nano-shell and optical coherence tomography agents. Additionally, positron emission tomography, computed tomography analysis, and single photon emission computed tomography employ gold nanocarriers [5].

Magnetic Nanocarriers

Nanocarriers with magnetic properties usually have a magnetic core that makes up the magnetic nanocarrier. In general, metal NPs exhibit greater magnetic properties than their metal oxide counterparts. Its magnetic and tailored characteristics make it suitable for biosensing applications. Superparamagnetic NPs exhibit heightened responsiveness to magnetic fields compared to paramagnetic NPs. Because of this characteristic, polymer-coated NPs of superparamagnetic iron oxide are frequently used as contrast agents in biochemical imaging methods. They also enhance particle and cell internalization. The paramagnetic properties diminish when the magnetic fields are no longer present. Furthermore, superparamagnetic iron oxides can passively target cancer cells. Magnetic NPs, due to their surface functionalization, can be used as sensors in implant components reliant on magnetic resonance imaging. Examples of magnetite NPs include ferric oxide, gamma iron oxide, and nanoscale ferrites. Because of its special qualities, magnetic NPs can be used as contrast agents, hyperthermia mediators, and targeted medication and gene treatment. An attempt to connect the medicine epirubicin to ferrofluid led to the drug's buildup at the intended location [14].

Mesoporous Silica

Because of its enormous porous honeycomb structure, mesoporous silica allows for the integration of more drug molecules. Its availability and ease of use make it incredibly useful in the biological field. For targeted medication delivery, these NPs can encapsulate both hydrophilic and hydrophobic medications that have the ability to bind to molecules of ligands. Mesoporous silica is distinguished by its extensive surface area, pore volume, biocompatibility, ability to load drugs efficiently, and thermal and chemical stability. This mesoporous silica effectively enables both active and passive targeting in cancer treatment. For instance, mesoporous silica is an effective way to administer anticancer medications such as methotrexate and camptothecin [13].

Hybrid nanocarriers

Nanocarriers that combine two or more inorganic and organic nanocarriers either singly or collectively are known as hybrid nanocarriers. It consists of multiple, inorganic-inorganic, and organic-inorganic components. Lipid-polymer hybrids and ceramic-polymer hybrids are a few instances of hybrid nanocarriers. When two NPs are combined, their dual natures will be present, greatly boosting their respective properties. Organic nanocarriers, such as liposomes, have poor stability and internal solution leakage problems. This facilitates its easy extraction from the bloodstream. Therefore, it can be used for medication administration with further stabilization [14]. The hybrid nanocarrier system compensates for those drawbacks. The choice of nanocarriers is influenced by factors including the target site, the specific drug for conjugation, physiological challenges during drug administration, and the durability and permeability of the small molecules. The primary objective of choosing appropriate nanocarriers for medication administration is to maximize the medication's absorption while reducing side effects. Several research studies have explored dual-component nanocarriers, including the mesoporous silicon dioxide NP-lipid bilayer system, which has demonstrated effective intracellular transport of zoledronic acid is utilized in the management of breast cancer to address bone metastases and associated complications, highlighting its maximum retention rates. This approach prevents premature drug release into the body by enabling a stimuli-responsive release of the medicine. It has been discovered that novel albumin hybrid nanocapsules are effective at encasing hydrophilic peptides or other tiny therapeutic molecules that are intended to target cancer cells. It permits decreased toxicity and even distribution in the tumor cells’ microenvironment. In drug delivery applications, ferritin offers a notable advantage by encapsulating therapeutic agents and facilitating stimulus-responsive release, ensuring sustained drug release at the targeted location. Numerous studies have explored the delivery of small interfering RNA (siRNA) in vivo using poly(amidoamine) hybrid nanocarriers. These nanocarriers include formulations like PEG-liposome/siRNA small particles and peptide HAIYPRH, referred to as siRNA small particles, which incorporate cholesterol and lipid core/shell configurations. This approach is effective in maintaining long-term stability and strong cellular uptake [11].

Foundational elements of Quality by Design (QbD)

**Figure 7:**
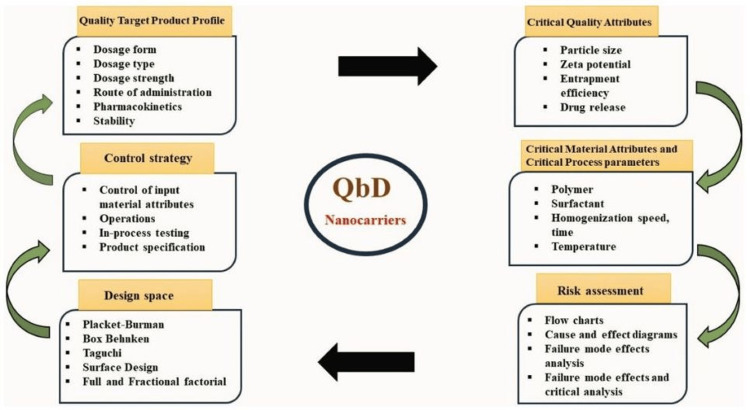
Element of Quality by Design approach This figure is created by the author, Karthick Rajan S. QbD, Quality by Design

In the production of pharmaceutical PEGylated nano-formulated lipid carriers, an applicant employs the QbD approach to pinpoint essential patient-centric vital product specifications. This assists in recognizing the product characteristics and translating them into critical quality attributes (CQAs) for pharmaceutical products. The Quality Target Product Profile (QTPP), critical material attributes (CMAs), CQAs, critical process parameters (CPPs), design space, process tools for analysis (PAT), control strategy, and risk assessment are the principal components of QbD. The element of QbD approach is shown in Figure 7.

QTPP

The QTPP, which also helps identify the product's CQAs, is a predefined future-oriented summary of the therapeutic product traits required to maintain the desired quality in terms of safety and therapeutic outcomes. QTPP ultimately acts as a link, aligning patient expectations with product excellence. For the creation of QTPP, the relationship between the product's physicochemical properties and biological performance as the goal product profile must first be determined [19]. The delivery of PEGylated nano lipid carriers to specific cells, tissues, or organs, along with the pharmacokinetics of the intended formulation, its toxicological characteristics, and the safety and effectiveness profile of these carriers, are typically associated with the vital biological activity qualities of a nano lipid formulation. Different laboratory and animal-based disease models can be employed to assess the safety and clinical benefits of specifically formulated nano lipid formulations. The QTPP for developing nanocarriers includes considerations such as active ingredient dispersion behavior, pharmacokinetic behavior, stability duration, purity and sterility parameters, packaging and sealing solutions, dosage form (e.g., freeze-dried powder or suspension for oral formulations, injectable formulation, gel, or topical cream for topical preparations), and potency level (benefiting from improved bioavailability and controlled drug release). These factors collectively define the desired attributes and performance expectations of nano lipid carrier formulations [8,12]. The target product profile for nanotechnology-based formulations is listed in Table 6.

**Table 6:** Target product profile for nanotechnology-based formulations API, active pharmaceutical ingredient

Quality Target Product Profile	Goal	Reasoning
Pharmaceutical form	Nanoparticle formulations	To enhance the effectiveness, permeability, durability, and absorption rate of therapeutic agents, depending on the specific medication and preparation.
Delivery route	Topical, oral, or injectable applications	Eye-friendly, local application with minimal invasion and minimized side effects.
Appearance	Freeze-dried powder or gel formulations	Take into account the final product's visual appeal to make management easier.
Physiochemical characterization	Efficiency of encapsulation	To guarantee effective medication loading in nanotechnology.
	Particle dimension	Affect permeation and adsorption.
	Electrokinetic potential	Impact on product stability.
	Release of the drug	Effect on the pharmacokinetics of the drug.
API solubility in carrier system (drug loading)	Up to 50% (indicating a high percentage)	Impact on drug dissolution profile, dermal penetration rate, and clinical efficacy.
Distribution	100-500 millipascal seconds	Impact on drug dissolution and durability.
Acid-base balance	Compatible with the formulation	Impact on drug liberation, drug entrapment, absorption rate, and durability.
Container system	Appropriate for the dose form	In order to guarantee the intended lifespan.
Durability	Quality standards	Effect on the standard of the product.
Pharmacokinetics	Absorption, distribution, metabolism, and targeting (ADMT)	Essential for achieving the desired efficacy.

CQAs

The QTPP defines CQAs, which direct both process and product development. These CQAs also set the criteria or limits within which quality products are accepted to maintain the desired standard of the nano-products. According to ICH Q8(R2), CQAs are the physical attributes, chemical properties, and biological characteristics of a drug product that must fall within defined limits to confirm the intended level of standard. CQAs, alongside the physical properties (like color, particle size, shape, and formulation appearance), chemical attributes (including entrapment efficiency, analysis, drug liberation profile, residuals analysis, robustness, and organic solvent residues, if applicable, and microbial attributes (encompassing pharmacokinetics, pharmacodynamics, and sterility), establish a connection between product quality and therapeutic effectiveness [4]. CQAs are commonly linked to the medication component, in-process ingredients, formulation excipients, and process variables. These CQAs are screened after being extracted from QTPP according to their risk or criticality regarding patient safety and product efficacy. They are used in risk assessment, drawing from past experience or established databases, to establish CMAs and CPPs [11].

CMAs

**Figure 8:**
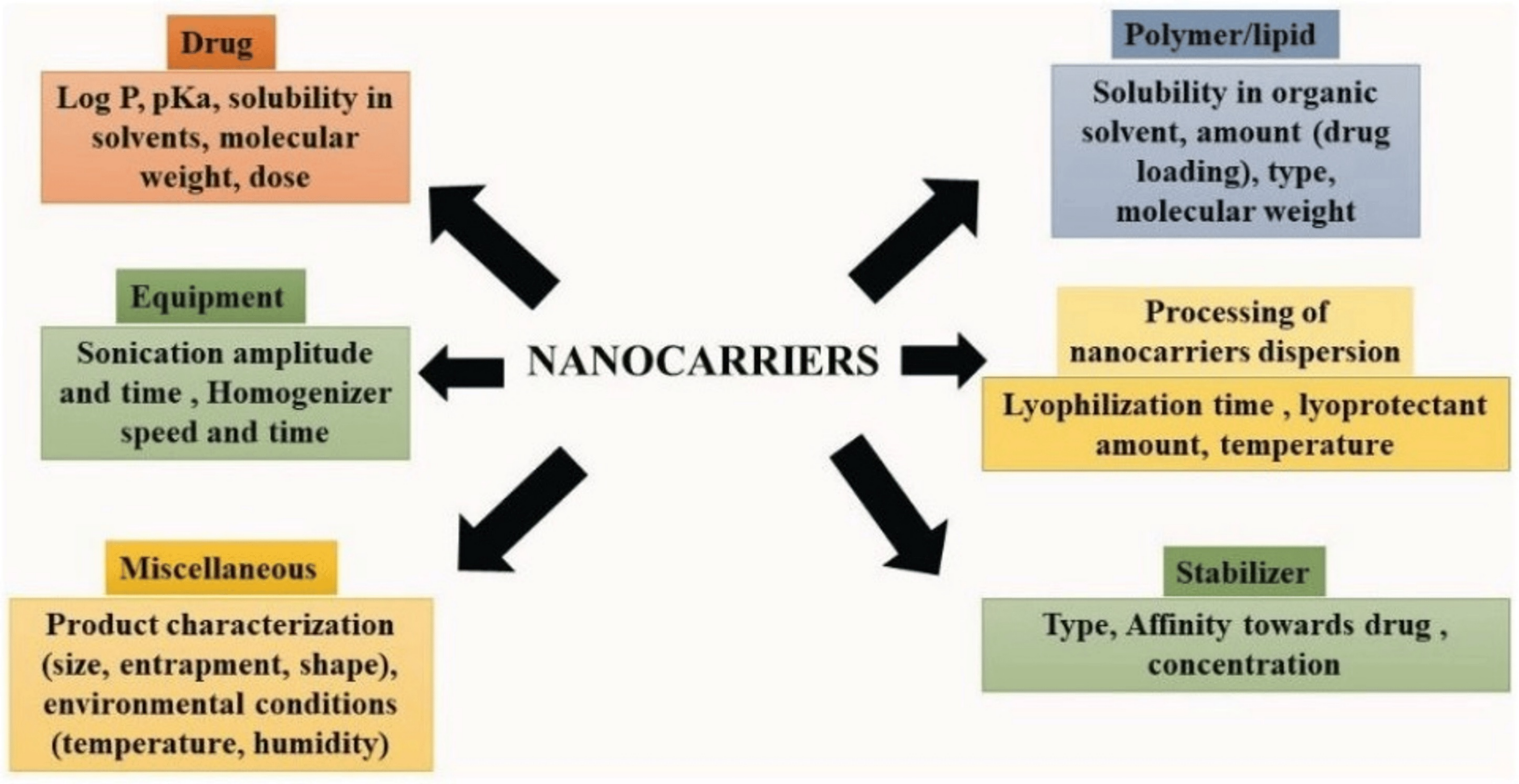
Various factors in formulation and processing may have an effect on the critical quality attributes (CQAs) of nanoformulations, influencing their effectiveness and safety This figure is created by the author, Karthick Rajan S.

The first group of variables that can affect the variability of CQAs are CMAs, which have to do with the nanocarrier's composition and preparation. To guarantee the finished product's required quality, CMAs - encompassing material properties and biotherapeutic or microbiological attributes of raw materials - must adhere to acceptable standard limits. Examples of CMAs in nanocarriers include surfactant level, variety, and quantity; level of organic solvent (for processes such as solvent evaporation); polymer and lipid levels and composition; as well as the kind and concentration of salts employed to prepare the buffer or give particles charge [19]. CQAs are crucial features that must be managed during the product development process in order to guarantee the safety, effectiveness, and quality of the finished product. Particle size, purity, stability, and chemical composition are a few examples of these characteristics. They work together to affect the manufacturability, safety profile, and performance of the product. Particle size, for example, can influence a drug's distribution and absorption, whereas purity affects safety and effectiveness. In order to ensure consistency and conformity with regulatory standards, controlling CQAs entails rigorous monitoring and adjustment throughout the manufacturing process, utilizing strong quality control techniques and validation protocols. Various factors in formulation and processing may have an effect on the CQAs of nanoformulations, influencing their effectiveness and safety, as shown in Figure 8.

CPPs

The second group of variables that can influence CQA variability is known as CPPs, which relate to the manufacturing process of nanoformulations. The input variables need to be regulated within predetermined ranges or limits in order to guarantee superior product quality are included in CPPs. These process factors affect both CQAs and the QTPP. Examples of CPPs for nanocarriers include the sequence of phase incorporation, temperature stabilization, manufacturing volume, blending rate and duration, ultrasonic speed, rotation speed and duration, among others [6].

Risk Assessment

The primary objectives of hazard assessment are to identify the CPPs of the drug product and assess the influence of specific variables or essential attributes of fundamental materials, active substances, additives or inert substances, and container components. Based on how each attribute might affect the product, it is categorized as either a low, medium, or high risk. We look into high-risk features more to lower the chance of risk. A biotechnology business used a number of risk assessment techniques when creating a medicine delivery system based on NPs to make sure it was safe and effective [19]. They implemented control measures to stop problems such NP aggregation by using failure mode and effects analysis (FMEA) to detect and address these difficulties. Critical hazards, such as poisonous metal pollution, were given priority in the risk ranking, which resulted in strict quality standards. Several scenarios were modeled using Monte Carlo Simulation in order to maximize the efficiency of medication delivery and NP size. Tests of different synthesis conditions and fine-tuning of NP properties were conducted using Design of Experiments (DoE). Ultimately, the business ensured regulatory compliance and openness by documenting and informing all relevant parties of all findings [19]. This all-encompassing strategy guaranteed the quality of the finished product and successfully handled hazards. Below is a summary from ICH Q9 of a non-comprehensive list of typical tools used in risk management:

(1) Ishikawa fishbone diagrams, flowcharts, check sheets, and other basic methods of risk management facilitation;

(2) fault tree analysis;

(3) risk rating and filtering;

(4) preliminary hazard analysis;

(5) hazard analysis and important control points;

(6) FMEA;

(8) operational analysis of hazards;

(9) auxiliary statistical instruments.

The kind of nano-formulation and the body of scientific information pertinent to the product under particular circumstances determine which particular instruments should be chosen [4]. The reasoning behind the initial evaluation of material characteristics and risks is listed in Table 7.

**Table 7:** Reasoning behind the initial evaluation of material characteristics and risks

Critical Quality Attributes of Drug Product	Critical Material Attributes	Reasoning
Polydispersity index	Pharmaceutical compound	Size is not significantly affected by drug characteristics when they are dissolved or distributed in phospholipid or polymer solutions.
	Lipid-based solid carriers	Size variations may occur due to changes in lipid concentration. If lipid utilization is incomplete (such as when using higher liquid volumes), particle size may increase.
	Fluid lipid	Liquid lipids have little effect on small carriers' size.
	Surfactant level	The surfactant is vital for minimizing size by stabilizing nanocarriers and averting particle aggregation.
Drug loading efficiency	Pharmaceutical compound	Entrapment is influenced by the characteristics of the substance; medicines that are extremely lipophilic are more likely to encapsulate.
	Lipid-based solid carriers	Reaching the ideal lipid concentration is essential to improving capture effectiveness.
	Fluid lipid	Liquid lipids improve the drug's encapsulation effectiveness.
	Surfactant level	Surfactants have minimal impact on drug entrapment.
Drug release	Pharmaceutical compound	The attributes of the drug moderately influence drug release.
	Lipid-based solid carriers	Lipids regulate drug release through encapsulation, enabling controlled drug delivery.
	Fluid lipid	The concentration of liquid lipids moderately affects entrapment efficiency.
	Surfactant level	The surfactant has a minimal effect on drug liberation.
Electrokinetic potential	Pharmaceutical compound	The drug minimally affects the zeta potential.
	Lipid-based solid carriers	The charge of the lipid influences the zeta potential to some extent.
	Fluid lipid	Liquid lipid minimally affects the electrokinetic potential.
	Surfactant level	In addition to imparting charge, the stabilizer is essential in defining the electrokinetic potential.

Regulatory status and safety profile of nano lipid carrier

Regulatory bodies are typically aware of natural phospholipids. Their remarkable multifunctional role is further enhanced by their excellent biocompatibility and extremely high tolerance. The WHO does not impose any restrictions on the oral intake of lecithin components. Additionally, there as a food ingredient, lecithin has no upper restriction on the quantity of dairy products that can be consumed. The FDA has released the Federal Register (FR) listing substances and compounds GRAS, along with an additional list. The IIG provides a list of ingredients in pharmaceuticals that have received FDA approval and are employed in marketing goods. Adjuncts play a crucial role in the formulation of medications, and understanding their properties and effects is essential before beginning the formulation process [8]. This is unquestionably important given the unique physicochemical properties of lipid excipients and their potentially intricate interactions with other elements in formulations or in vivo biological settings. Previously, it was assumed that volume expanders, cohesive agents, packing agents, anti-friction agents, shell materials, liquid carriers, and dyes were inert and did not interact with the biological system, the drug compound, or the packaging. However, recent studies have revealed a wide range of novel non-active substances with diverse toxicity profiles and interactions. Therefore, evaluating the harmful effects and compatibility characteristics of fatty additives meant for use in formulations is crucial. Two critical considerations in product development. Regardless of how effective a drug transportation system may be, using it in a clinical environment is not feasible if any of its components present risks. Fortunately, because triglycerides are widely found in daily foods and the human body, lipid safety is rarely a problem. The vast majority of lipids are GRAS. However, the safety of lipid formulations can be influenced by the inclusion of co-surfactants or cytotoxic surfactants. Therefore, it is advisable to use GRAS-approved surfactants when manufacturing various phospholipid formulations. The administration routes of fatty additives must be taken into account while assessing their regulatory status. Most fatty excipients are going to be used in topical and the creation of cosmetic products. For essential lipids, it is deemed safe to use lipid excipients in dermal and oral applications of several formulations based on lipids such as lipid solid small particles, nanostructured fatty carriers, and others [19]. A specific lipid type cannot be directly used in pharmaceutical formulations due to its existing use in the food industry. However, a novel lipid's toxicity data can be used to get approval from pharmaceutical organizations for food usage. Lipids are substances from the food business that can be used to make pharmaceuticals after receiving regulatory approval. Although nanostructured colloidal lipid formulations and solid-state lipophiles (SLNs) are considered safe and do not pose any toxicity concerns, they are not the recommended carrier for parenteral drug administration. Nevertheless, the class of lipids used in parenteral medication delivery is distinct from the categories used in lipid-based formulations. Parenterally administered liposomes have been used for a long time to treat a wide range of disorders, and there are no negative side effects [8].

Future perspective

To enhance the biocompatibility of many colloidal carriers, PEGylation is an essential step. It offers at least some in vivo benefits in the great majority of cases, making it a great place to start for researchers working on the development of nanocarriers. This has made it possible to carry out research in this crucial field of nanomedicine, which has a bright future ahead of it. PEGylation as a formulation method has led to the release of some novel medications that are bettering patient care by decreasing toxicity or requiring fewer doses. However, in order to keep the field of nanomedicine moving forward, we need to keep refining PEGylation and searching for new and better ways to deliver nanocarriers Above all, more effective characterization techniques need to be created and widely applied. Numerous characterization techniques exist, but each has its limits. While some only offer basic qualitative evaluations, some also offer quantitative evaluations. These could show where PEG is located within the particle, but it's still hard to say where PEG is homogeneous on the surface. Assays conducted both in vivo and in vitro are frequently costly and time-consuming, yet differences between the outcomes of these experiments are nevertheless seen. Therefore, it is extremely desired to develop a method that would enable a quick and affordable evaluation of PEG on particle surfaces and whose outcomes would be consistent with in vivo success. Secondly, it might be essential to advance PEGylation techniques beyond modifications to PEG chains' length and shape or techniques for affixing PEG to colloidal carriers. These small changes have improved plasma half-life and reduced RES system uptake, but they still need improvement. Increasing the flexibility of PEG molecules is one area that is presently being investigated. Computer models have revealed that more flexible polymers have more conformations and quicker rates of transition between different conformations. Thus, opsonin penetration is inhibited by more flexible polymers that are able to build a denser and more homogenous water cloud. This suggests that PEG's stealth-enhancing qualities could be enhanced by any increases in its flexibility. This can be achieved by altering the structure or by utilizing various solvents. For example, it has recently been demonstrated that the adsorption of CO2136 by supercritical carbon dioxide (SC-CO2) as a solvent increases the volume of a PEG1500 chain and lowers its melting point, suggesting an increase in chain mobility. Increasing the volume and chain mobility together can have a significant impact on the repulsive qualities of proteins. Conversely, it might be required to modify the nanocarriers themselves in order to combine fresh enhancements with those previously observed through PEGylation. It has been stated that smart medication delivery systems have been developed, and their significance is expected to grow. These methods minimize release until the intended localization. Article form, which includes the utilization of asymmetric NPs, is another feature that has received little research but, with proper optimization, could lead to better drug delivery. Non-spherical particles may have an impact on and maybe enhance targeting, internalization, degradation characteristics, and travel through the body. Although PEGylation of surfaces is not without its problems, it appears that it will remain the dominant method of imparting stealth properties to nano-lipid carrier drug delivery systems for the foreseeable future. Anti-PEG antibodies can lessen the efficiency of PEGylation and trigger immunological responses, despite the fact that it is widely used to improve drug stability and circulation. In order to solve this, we need to look into PEGylation substitutes, research the effects of these antibodies on medications, assess changes in the market and regulations, and keep a watch on emerging technologies. This strategy will contribute to offering a more thorough picture of PEGylation's future.

## Conclusions

The pharmaceutical industry has been concentrating more on polymer-drug combinations in the last few decades, which has led to a lot of research and development. Bioresorbable polymers, novel linked compounds, sophisticated drug targeting, and effective synthesis methods are important research fields. One well-known method that increases medication solubility, stability, and circulation time while reducing adverse effects is called PEGylation. This has significantly improved patient outcomes for a variety of illnesses by advancing biologics and other medicines. Because PEGylated systems prolong medication half-lives and improve bloodstream availability, they are especially useful in treating metabolic diseases, blood coagulation disorders, oncological disorders, and persistent pain syndromes. Nonetheless, obstacles, including exorbitant manufacturing expenses and possible immunological reactions, endure. Investigating substitute polymers, minimizing immunological responses, and cutting expenses are necessary to address these issues. PEGylated systems enable higher concentrations of active components and better treatment targeting in oncology. By improving accuracy and dosage, developments in PEGylated polymer-drug conjugates also hold promise for the treatment of hemophilia and diabetes. Polyamides for implants and long-term release, biodegradable polymers like PLA and PGA for tissue scaffolds and controlled-release formulations, and polysaccharides like hyaluronic acid and chitosan for targeted and oral delivery are just a few of the possible advantages of various polymers. These approaches show the continual evolution of medication delivery and the hunt for novel solutions to PEGylation.
